# *Rhizosphere* bacteria associated with *Chenopodium quinoa* promote resistance to *Alternaria alternata* in tomato

**DOI:** 10.1038/s41598-022-21857-2

**Published:** 2022-11-08

**Authors:** Sidra Zahoor, Rabia Naz, Rumana Keyani, Thomas H. Roberts, Muhammad N. Hassan, Humaira Yasmin, Asia Nosheen, Saira Farman

**Affiliations:** 1 Department of Biosciences, COMSATS University Islamabad, Park Road, Chak Shahzad, Islamabad, Pakistan; 2 School of Life and Environmental Sciences, University of Sydney, Sydney, Australia; 3 Department of Biochemistry, Abdul Wali Khan University Mardan, Mardan, Pakistan

**Keywords:** Microbiology, Plant sciences

## Abstract

Microorganisms can interact with plants to promote plant growth and act as biocontrol agents. Associations with plant growth-promoting rhizobacteria (PGPR) enhance agricultural productivity by improving plant nutrition and enhancing protection from pathogens. Microbial applications can be an ideal substitute for pesticides or fungicides, which can pollute the environment and reduce biological diversity. In this study, we isolated 68 bacterial strains from the root-adhering soil of quinoa (*Chenopodium quinoa*) seedlings. Bacterial strains exhibited several PGPR activities in vitro, including nutrient solubilization, production of lytic enzymes (cellulase, pectinase and amylase) and siderophore synthesis. These bacteria were further found to suppress the mycelial growth of the fungal pathogen *Alternaria alternata*. Nine bacterial strains were selected with substantial antagonistic activity and plant growth-promotion potential. These strains were identified based on their 16S rRNA gene sequences and selected for *in planta* experiments with tomato (*Solanum lycopersicum*) to estimate their growth-promotion and disease-suppression activity. Among the selected strains, *B. licheniformis* and *B. pumilus* most effectively promoted tomato plant growth, decreased disease severity caused by *A. alternata* infection by enhancing the activities of antioxidant defense enzymes and contributed to induced systemic resistance. This investigation provides evidence for the effectiveness and viability of PGPR application, particularly of *B. licheniformis* and *B. pumilus* in tomato, to promote plant growth and induce systemic resistance, making these bacteria promising candidates for biofertilizers and biocontrol agents.

## Introduction

The rhizosphere is a major site for a diversity of microbial interactions with plants. Members of the microbial community in the rhizosphere may be beneficial or non-beneficial for plant growth and development^1–3^. Some microbes (soil-borne pathogens) have deleterious effects on plant health through infection and competition with beneficial microbes and the plant for nutrients. Other microbes like plant growth promoting bacteria (PGPB) and some mycorrhizae-forming fungi promote plant growth by mitigating stresses caused by biotic and abiotic factors, promoting nutrient mobilization and enhancing yield^4,5^. Specific compounds in root exudates mediate signaling interactions with microbes. Metabolites including sugars, amino acids and organic acids, as well as various other carbon compounds, are delivered to the rhizosphere to support microbial growth^6–8^.

Root-colonizing rhizobacteria are reported to confer beneficial impacts on plants through direct mechanisms such as ammonia production and regulation of phytohormone levels, as well as nutrient solubilization, but indirect mechanisms like production of HCN, siderophores and antibiotics are also involved^9^. Many bacterial species, including those belonging to the genera *Bacillus, Azotobacter, Azospirillum, Klebsiella, Pseudomonas, Alcaligenes, Burkholdeira, Arthrobacter, Serratia* and *Enterobacter*, are reported to promote plant growth and can be categorized as plant growth-promoting bacteria (PGPB)^7,9–11^. In tomato, rhizobacteria including *Bacillus* spp*., Azotobacter* spp*., Pseudomonas fluorescens, Micromonospora* spp. and *Serratia* spp.^12–15^ are reported to influence plant-pathogen interactions and stimulate plant growth.

One of the most common fungal pathogens of tomato is *Alternaria alternata*, which causes disease characterized by the presence of early blight, stem and fruit canker^16^. Early blight caused by *A. alternata* has great economic importance globally. This disease reduces the photosynthetic surface of the leaves and, in severe cases, leads to complete plant defoliation^17^. According to a field survey conducted in 2016–2017 in different regions of Pakistan, *A. alternata* was found to be the second most common pathogen isolated from leaves and fruits of tomato, with disease incidence ranging from 9 to 75%^18^. The main meteorological components contributing to pathogen invasion and deterioration of a tomato crop are temperature and relative humidity*.* Strictly speaking, *A. alternata* is a necrotrophic pathogen but, in the case of severe damage to the plant, it can also be transmitted to the seeds, thereby affecting the next generation^19^. *A. alternata* is reported to produce specific fungal toxins called AALs (an abbreviation for *A. alternata* f. sp. *lycopersici*)^20^. These host-selective toxins (HST) lead to apoptosis followed by necrotic spots on leaf, stem and fruit^21^.

Biological control of plant infectious diseases prevents collateral damage to beneficial microbes caused by the use of synthetic fungicides^22^. Biofertilizers and biopesticides are both environmentally friendly and potent for fungal control and are reported to promote the availability of soil nutrients to plants. In this respect, the use of plant growth-promoting rhizobacteria is considered one of the most effective approaches to control pathogens^23^. Bacterial strains from the order Bacillales have been particularly useful^24–26^ and are commonly commercialized for these applications^27^. Strains of the *Bacillus* genus are recognized for their antagonist activity against tomato pathogens^28,29^. The biocontrol activity of these bacteria has been attributed to their ability to produce antibiotic compounds and to compete for space and nutrients in the rhizosphere^30^. Chowdappa et al.^31^ reported that strains of *B. subtilis* inhibited the growth of mycelium from *A. alternata*, *A. solani* and *P. infestans *in vitro. In tomato plants, the inoculants induced the production of auxins and gibberellins and increased the activities of peroxidase, polyphenol oxidase and superoxide dismutase, which contributed to systemic resistance in tomato plants against early and late blight.

In the present study, the rhizosphere of the quinoa plant (*Chenopodium quinoa* Willd), which belongs to the large family Amaranthaceae, was explored to identify potent biocontrol agents. Quinoa is a native crop of the Andean Plateau in west-central South America, and was introduced to Pakistan recently for its revenue-generating potential, its higher adaptability to climatic conditions and potential for improvement in the cropping system^32–35^. Quinoa is renowned for its ability to withstand drought and salinity^36^ and for its remarkable nutritional value^37^.

The objectives of this research were to (1) isolate and identify beneficial rhizobacteria associated with *C. quinoa*; (2) evaluate the growth-promoting and fungal pathogen-antagonizing potential of these rhizobacteria; (3) evaluate the biological control ability of the rhizobacteria against *A. alternata *in vitro and in vivo, and (4) investigate the effects of exogenous inoculations on the antioxidant defense system of tomato in the presence of *A. alternata*.

## Materials and methods

### Crop location, growth conditions and rhizosphere sampling

Cultivated *Chenopodium quinoa* plants along with roots and rhizospheric soil were collected and identified by the National Herbarium, Department of Plant Sciences, Quaid-i-Azam University, Islamabad. Plant collection was done from two districts of Pakistan, Khanewal (30.2864° N, 71.9320° E) and Faisalabad (31.4504° N, 73.1350° E), which are considered irrigated climatic zones because of their inclusion in the sugarcane belt. Annual precipitation of the sampling year was recorded as 202.78 mm and 347.2 mm for Khanewal and Faisalabad, respectively. The soil in the rhizosphere was characterized as a clay loam. Samples from the rhizosphere of quinoa plants were obtained at a depth of 20–30 cm. Soil and root samples weighing approximately 50 g were collected from the individual area of the field, stored in sterile plastic bags on ice, taken to the laboratory immediately, and stored at 4 °C.

The plant material collected and used in this study completely complies with institutional, national, and international guidelines and legislation regarding this type of experiment. Moreover, permissions were obtained from agricultural farms based in Khanewal and Faisalabad**,** for collecting and using the plant in this study.

### Bacterial isolation and differentiation

To isolate rhizosphere bacteria, the soil in the immediate vicinity of the quinoa roots was carefully isolated, crushed and sieved under aseptic conditions. Serial dilutions of the soil were made using sterile 0.8% saline solutions. Aliquots (100 μL) of each serial dilution were spread on LB as well as on nutrient agar media plates and incubated at 28 ± 2 °C for 24–48 h. Colony forming units were counted on the following day and representative colonies exhibiting different morphological aspects were differentiated, spotted and isolated to form pure cultures. Bacterial cultures were differentiated and purified by observing their color, texture, margins, optical density, size and shape. Overall, 68 different colonies were isolated based on their colony morphology and were then subjected to gram staining (Supplementary Table 1).

### In vitro screening for antagonistic activity

All the isolated bacterial strains were subjected to in vitro testing for their antagonistic activity against *Alternaria alternata* cultures obtained from the Applied Microbiology and Biotechnology Lab of COMSAT University Islamabad. The bacteria were grown for 24 h in LB and/or nutrient broth according to their isolation media at 28 ± 2 °C. An aliquot (70 µL) was taken from the overnight-grown bacterial cultures with 10^8^ CFU/mL and spread uniformly on PDA plates. A 5-mm plug of 7 days-old *A. alternata* culture was placed in the middle of the plate and incubated at 30 °C for 7 days. Control plates without bacterial inoculation were also prepared. The percentage growth inhibition was measured by comparing mycelial growth with control plates and calculated using the following formula:

$$\% \;{\text{inhibition}} = \left[ {({\text{C}} - {\text{T}}) \times {1}00)/{\text{C}}} \right]$$

where C is the growth of fungal mycelia in control plate, and T is the growth of fungal mycelia in bacteria-inoculated plates.

### Production of extracellular enzymes and siderophores

The selected strains based on their antagonistic potential were further assessed for their potential to produce major antifungal enzymes and siderophores. Cellulase production was detected by testing bacteria on carboxymethyl cellulose (sodium salt) (CMC) agar^38^, whereas detection of siderophore production was facilitated using chrome azurole sulphur (CAS) agar^39^. The CMC and CAS agar plates were inoculated with 24 h freshly grown cultures by the spot inoculation method and incubated at 28–30 °C for 4–7 days. Orange-colored halo zones around the bacterial colony were observed in CAS agar plates and their diameters measured in millimeters. Cellulase production was observed by staining with 0.1% congo red solution and destaining with 1 M NaCl solution.

For pectinase and amylase activities, plates containing pectin^40^ and 1% soluble starch^41^ were prepared and inoculated with 24-h bacterial cultures. After incubation for 4–7 days, the plates were flooded with 1% lugol solution (10 g KI, 5 g iodine, 100 mL distilled H_2_O). Decolorized clear halo zones were observed and their diameters measured in millimeters. All experiments were performed in triplicate.

### In vitro screening for plant growth-promoting traits

#### Phosphate, potassium and zinc solubilization assays

Selected bacterial strains were further screened using nutrient solubilization assays to assess their nutrient solubilization potential. The selected strains were tested for phosphorus, zinc and potassium solubilizing assays using Pikovskaya agar^42^, Burnt and Rovira agar^43^ and Aleksandrove agar^44^, respectively. All the selected bacterial cultures were streaked on the respective media to obtain 24 h fresh cultures. These bacteria were then inoculated on media plates by the spot method and incubated at 28–30 °C for 4–7 days. The presence of a halo zone around the bacterial colony in the respective agar medium showed their potential to solubilize the nutrient. The zone and colony dimensions were measured in millimeters. The solubilization index was calculated using the following formula:

$${\text{Solubilizing}}\;{\text{Index}} = \left( {{\text{Colony}}\;{\text{diameter}} + {\text{Halo}}\;{\text{zone}}\;{\text{diameter}}} \right)/{\text{Colony}}\;{\text{diameter}}$$

### Petri plate experiment

In vivo growth promotion activity was estimated via a petri plate experiment. Tomato seeds (Rio grande) were obtained from the National Agriculture Research Centre (NARC) in Pakistan. The selected bacterial cultures were grown in LB broth or nutrient broth according to their isolation medium. The OD of 24 h grown bacterial cultures was maintained at 1.0. The cultured broth was centrifuged at 10,000 rpm for 5 min and bacterial pellets were resuspended and dissolved in autoclaved distilled water. Tomato seeds, which were surface-sterilized with 3% sodium hypochlorite (NaOCl) solution and 70% ethanol for 1 min, separately, and washed 4–5 times in autoclaved distilled water^45^, were soaked in bacterial suspension for 3 h. The bacteria-treated seeds were sown in sterile petri plates fitted with a layer of cotton and sterile filter paper. The experiment was conducted in triplicate and seeds treated with distilled water were characterized as the control. Germinated seeds were observed and counted on a daily basis up to 15 days from sowing to calculate the germination percentage, promptness index, emergence index and vigor index, using the formulas below^46^. Difference in seedling length and fresh weight was also measured. All treatments were performed in triplicate.

$$\begin{aligned} & {\text{Germination}}\;{\text{percentage}} = {\text{Number}}\;{\text{of}}\;{\text{seeds}}\;{\text{germinated}}/{\text{Total}}\;{\text{number}}\;{\text{of}}\;{\text{seeds}} \times {1}00 \\ & {\text{Promptness}}\;{\text{index}} = {\text{nd}}_{{2}} \left( {{1}.00} \right) + {\text{nd}}_{{4}} \left( {0.{75}} \right) + {\text{nd}}_{{6}} \left( {0.{5}0} \right) + {\text{nd}}_{{8}} \left( {0.{25}} \right) \\ \end{aligned}$$

where nd_2_, nd_4_, nd_6_ and nd_8_ is the number of seeds germinated on 2nd, 4th, 6th and 8th day, respectively.

$$\begin{aligned} {\text{Emergence}}\;{\text{index}} & = \left( {{\text{number}}\;{\text{of}}\;{\text{seeds}}\;{\text{germinated}}/{\text{days}}\;{\text{of}}\;{\text{first}}\;{\text{count}}} \right) + \cdots \\ & \quad + \left( {{\text{number}}\;{\text{of}}\;{\text{seeds}}\;{\text{germinated}}/{\text{days}}\;{\text{of}}\;{\text{final}}\;{\text{count}}} \right) \\ & {\text{Vigor}}\;{\text{index}} = {\text{S}} \times \Sigma \left( {{\text{Gt}}/{\text{Dt}}} \right) \\ \end{aligned}$$

where S is the length of the seedling measured on the 7th day after germination, Gt is the overall number of germinated seeds on the ‘t’ day, and Dt is the cumulative number of days from the initial to the ‘t’ day.

### Identification and phylogeny of selected PGPR

Genomic DNA of selected strains was extracted using the CTAB method^47^. Universal primers P1 (5′–CGGGATCCAGAGTTTGATCCTGGTCAGAACGAACGCT–3′) forward and P6 (5′–CGGGATCCTACGGCTACCTTGTTACGACTTCACCCC–3′) reverse^48^ were used to amplify the 16S ribosomal RNA gene. The PCR products were purified utilizing a PCR purification kit (Thermo Scientific) and sent to MCLAB (San Francisco, USA) for sequencing. The forward and reverse sequences were aligned using CLUSTALW. The aligned consensus sequences were subjected to BLAST on NCBI and EZ taxon^49,50^. The bacteria were identified based on their maximum sequence homology to the type strains^51^. After identification to check their phylogenetic relationship with other closely related strains, a phylogenetic tree was constructed using MEGA X.

### Pot experiment

The properties of selected PGPRs were further investigated in a pot experiment in the shade house at COMSAT University, Islamabad. Pots (18 cm diameter, 22 cm height) were filled with autoclaved sand, soil and peat moss in a ratio of 1:2:1. Tomato seeds were surface-sterilized with 3% NaOCl and 70% ethanol and subsequently washed (3×) with sterile distilled water. The seeds were then soaked in bacterial suspension maintained at 1.5 × 10^8^ CFU mL^−1^ for 3 h. Ten inoculated tomato seeds were sown in each pot. The experiment consisted of nine sets of treatments and two sets of positive and negative controls. The experiment was laid out in completely randomized design and conducted in triplicate.

### Pathogen inoculation

After 45 days of emergence, the tomato plants were inoculated with the *A. alternata* pathogen. For this purpose, a culture of *A. alternata* was grown in potato dextrose broth for 7 days at 28 °C in a shaking incubator. The mycelial sheet was separated by filtration and a spore suspension maintained at 1 × 10^5^ spores/mL by utilizing a hemocytometer. Tween 80 was added to the spore suspension at a final concentration 1% and the suspension used to both drench the soil near the plant root and spray onto the leaves^52,53^. The pathogen-inoculated plants were covered with polythene sheets for 24 h to maintain humidity^54^ and allowed to grow in a shade house at 26–28 °C during the day and 22–24 °C during the night.

### Sample collection

Leaf samples were collected on 3, 6 and 9 days after inoculation (dai), preserved in liquid nitrogen and stored at − 70 °C for further analysis.

### Disease scale and disease severity index

After 14 dai, plants were evaluated from all the replicates in random order to evaluate the degree of infection and the effectiveness of selected PGPR inoculations on the biocontrol of *A. alternata*. A disease score 0–5 was employed for the assessment of infection degree. A score of 0–1 indicated very early stage of infection with few symptoms; 1–2 indicated the appearance of brown spots and discolored regions around the spots; 2–3 indicated brown necrotic spots combined to form concentric rings, 3–4 represented an increase in the damaged area more than 50%, and 4–5 corresponded to severe damage when concentric rings join to form large patches of necrosis leading to wilting, discoloration and defoliation. Plants were evaluated according to this scale to calculate the disease severity index and percent disease reduction or disease protection for each treatment and control using the data from five replicates. The following formula was used to calculate the disease severity index as a percentage^55^:

$$\begin{aligned} {\text{DSI}}\;\left( \% \right) & = \left[ {{\text{sum}}\;\left( {{\text{class}}\;{\text{frequency}} \times {\text{score}}\;{\text{of}}\;{\text{rating}}\;{\text{class}}} \right)} \right]/\left[ {\left( {{\text{total}}\;{\text{number}}\;{\text{of}}} \right.} \right. \\ & \quad \left. {\left. {{\text{plants}}} \right) \times \left( {{\text{maximal}}\;{\text{disease}}\;{\text{index}}} \right)} \right] \times {1}00 \\ \end{aligned}$$

### Total soluble protein content

The total soluble protein content of leaves was determined for all sampled stages (3, 6 and 9 dai)^56^. Leaf tissue (0.1 g) was homogenized using 1 mL of sodium phosphate buffer (pH 7.5). The homogenate was centrifuged at 4000 rpm for 10 min and 0.1 mL of the supernatant transferred to a test tube to make the volume up to 1 mL with distilled water. The test tubes were then kept on a shaker for 10 min after adding 1 mL of CuSO_4_ reagent. An aliquot (100 µL) of Folin & Ciocalteu phenol reagent was added and the solutions allowed to incubate at room temperature for 30 min. The absorbance at 650 nm was recorded on a UV–visible spectrophotometer against sodium phosphate buffer as blank. The concentration of protein was determined by comparison to a bovine serum albumin (BSA) standard curve.

### Antioxidant enzyme activities

Leaf samples were homogenized at 4 °C to measure antioxidant enzyme activities using 0.1 M phosphate buffer (pH 6.8) for polyphenol oxidase (PPO) and peroxidase (POD), while borate buffer (pH 8.8) was used for PAL. The samples were centrifugated at 17,000*g* for 20 min and the supernatant characterized as enzyme extract, which was used for further reaction mixture preparations for each respective enzyme activity.

PPO and POD activities were determined using the method of Kar and Mishra^57^. A reaction mixture (total volume 1.5 mL) was prepared using 1.2 mL 25 mM phosphate buffer (pH 6.8), 250 µL 100 mM pyrogallol and 50 µL enzyme extract for the determination of PPO activity, while for POD activity 100 µM H_2_O_2_ was also included. The absorbance at 420 nm of triplicates from each treatment for POD and PPO activities was measured.

PAL activity was evaluated using the protocol of Peioxto^58^. A reaction mixture was made consisting of 200 µL 0.2 M borate buffer (pH 8.8), 200 µL of 50 mM phenylalanine and 20 µL enzyme extract. This mixture was incubated at 39 °C for 1 h. The reaction was stopped using 20 µL 6 N HCl and the absorbance at 290 nm determined.

### RNA extraction, cDNA synthesis and RT-qPCR

Total RNA was extracted from leaves using trizol reagent (Invitrogen). DNA contamination was removed using RNase-free DNase (TURBO DNAfree kit, Ambion, USA). RNA was quantified using a Colibro microvolume spectrophotometer (Titertek, Brethhold). cDNA synthesis was performed using an Oligo dT Maxima H Minus Double-Stranded cDNA Synthesis Kit (Thermoscientific) with single-stranded RNA as a template following the manufacturer’s instructions. Confirmation of cDNA synthesis was checked using conventional PCR with primers for the housekeeping gene *Actin*. Details of primers used for PR-1, β-1,3 glucanase, chitinase and PAL genes, as the internal control, actin, are given in Supplementary Table 1. All experiments were performed in triplicate with a negative control. Quantitative real time PCR (qRT-PCR) was performed using Fast SYBR™ Green Master Mix (Thermoscientific). The reaction mixture contained 2 µL of cDNA, 1 µL of forward and reverse primer (100 pm), 5 µL of Fast SYBR™ Green Master Mix and 1 µL of nuclease free water. PCR conditions used were 95 °C for 10 min for initial denaturation and 39 cycles of 95 °C for 15 s, 58 °C for 30 s, and 72 °C for 30 s.

### Statistical analysis

Data were expressed as mean ± standard error (n = 3) for each treatment. The values obtained for physiological and biochemical activities were subjected to analysis of variance (ANOVA) using Statistix v.8.1. Comparison between mean values of treatments to test significant differences at P ≤ 0.05 was made by least significant difference (Gomez and Gomez, 1984). Statistix v.8.1 was used to calculate Pearson’s correlation coefficients to determine the relationship between disease reduction and expression of each PR genes.

## Results

### Isolation and characterization of rhizosphere bacteria associated with *C. quinoa* antagonistic activity

**Figure 1:**
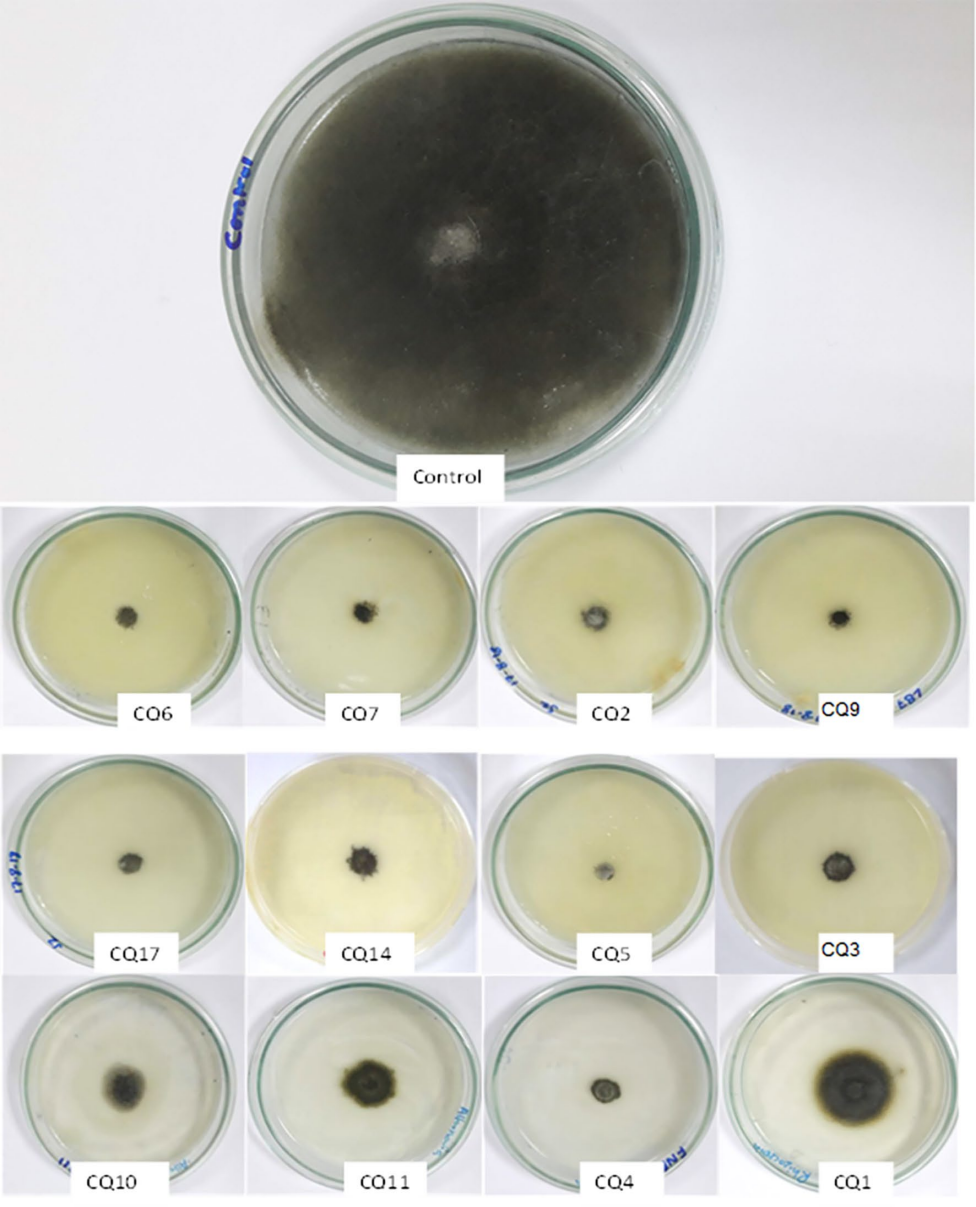
Antagonistic potential of selected bacterial strains against *Alternaria alternata*. Bacterial strains isolated from the rhizosphere of *C. quinoa* were subjected to antagonistic plate assay against *A. alternata*. The strains shown in this picture displayed maximum inhibition potential in addition to growth promoting activity and production of extracellular enzyme production, nutrient solubilization and siderophore production.After identification these strains were checked in pot experiment of tomato plants against *A. alternata* infection.

The 68 bacteria strains isolated from the *C. quinoa* rhizosphere were individually tested and screened for their antagonistic ability against *A. alternata *in vitro. All bacterial isolates were found to be antagonistic to the tomato pathogen, exhibiting values for percent inhibition ranging from 14 to 100% (Table 1). The isolated bacteria CQ5, CQ6, CQ7 and CQ9 exhibited inhibition of *A. alternata* growth by 100%, followed by CQ14 and CQ17 by 98%. The isolates CQ11, CQ2, CQ3, CQ4, CQ10 and CQ1 showed 88, 87.5, 87.5, 86, 80 and 77% inhibition, respectively, against *A. alternata* (Table 1, Fig. 1, Supplementary Table 2).

**Table 1:** Screening of rhizobacteria associated with *Chenopodium quinoa.*

Initial label	Modified label	Nutrient solubilization		Antifungal activity				Fungal antagonism
		Zinc	Phosphorus	Cellulase	Pectinase	Amylase	Siderophore	*A. alternaria* (%)
NA7	CQ1	3.81^c^	3.0417^a^	4.4523^cd^	3.6667^e^	0.00^g^	2.5667^h^	77^f^
NA8	CQ2	0.00^d^	0.00^f^	4.6811^c^	5.5667^ab^	3.7848^c^	3.4222^g^	87.167^d^
NA9	CQ3	2.889^c^	2.1513^e^	5.7806^a^	4.527^cd^	3.7435^c^	2.6548^h^	87^d^
NA10	CQ4	0.00^d^	2.6316^b^	4.6122^c^	5.0476^abc^	2.3144^f^	4.889^f^	86.9^d^
NA13	CQ5	0.00^d^	2.126^e^	6.1270^a^	4.9054^bc^	4.0389^b^	5.2857^e^	100^a^
LB6	CQ6	0.00^d^	2.5053^c^	3.8857^e^	4.8944^bc^	2.9792^d^	6.7063^b^	100^a^
LB7	CQ7	5.5^b^	2.35^d^	3.678^e^	2.9583^f^	0.00^g^	7.5556^a^	100^a^
LB9	CQ9	4.06^bc^	0.00^f^	0.00^g^	0.00^g^	0.00^g^	6.00^cd^	100^a^
LB10	CQ10	9.33^a^	2.2291^de^	5.5635^ab^	0.00^g^	0.00^g^	6.222^c^	80^e^
LB11	CQ11	2.822^c^	2.1830^e^	3.0019^f^	5.6190^a^	4.6175^a^	0.00^i^	88^c^
LB14	CQ14	3.556^c^	2.1417^e^	5.0119^bc^	2.6111^f^	0.00^g^	4.778^f^	98^b^
LB17	CQ17	0.00^d^	2.6349^b^	3.9352^de^	4.1141^de^	2.500^e^	5.8750^d^	98^b^

All values are means of three replicates. Values with different letters in the same column are significantly different at p ≤ 0.05.

### Nutrient solubilization

Twelve rhizospheric bacteria isolates were selected to assess their plant beneficial traits. Ten isolates displayed phosphate solubilization activity. The highest solubilization index was exhibited by CQ1 (3.04) while bacterial strains CQ3, CQ5, CQ6, CQ7, CQ10, CQ11, CQ14 and CQ5 exhibited a moderate solubilization index. In contrast, the bacteria CQ9 and CQ2 displayed no phosphate solubilization.

Among all the selected bacterial isolates, CQ10 (9.33) exhibited maximum zinc solubilizing ability, while CQ7, CQ9, CQ1, CQ14, CQ3 and CQ11 exhibited moderate to low zinc solubilization (Table 1). CQ2, CQ4, CQ5 CQ6 and CQ17 displayed no zinc solubilizing ability. None of the selected strains displayed potassium solubilization activity.

### Production of extracellular enzymes

The bacterial isolates were further examined for the production of extracellular enzymes (cellulase, pectinase, amylase) and siderophores. The solubilization index for cellulase enzyme production ranged from 3.0 to 6.13 for 11 of the strains while no cellulase production was detected using CQ9 (Table 1).

The solubilization index for pectinase ranged from 2.61 to 5.62 for 10 of the strains, whereas CQ9 and CQ10 displayed no pectinase activity (Table 1).

The solubilization index for amylase production ranged from 2.5 to 4.7 for CQ11, CQ5, CQ2, CQ3, CQ6 and CQ17. In contrast, no amylase production was observed in CQ1, CQ7, CQ9, CQ10 and CQ14. The solubilization index for siderophore production ranged from 2.56 to 7.56 for 11 of the strains, whereas no siderophore production was observed in CQ11 (Table 1).

### Effects of selected bacterial strains on tomato plant growth in Petri plate experiment under axenic conditions

**Figure 2:**
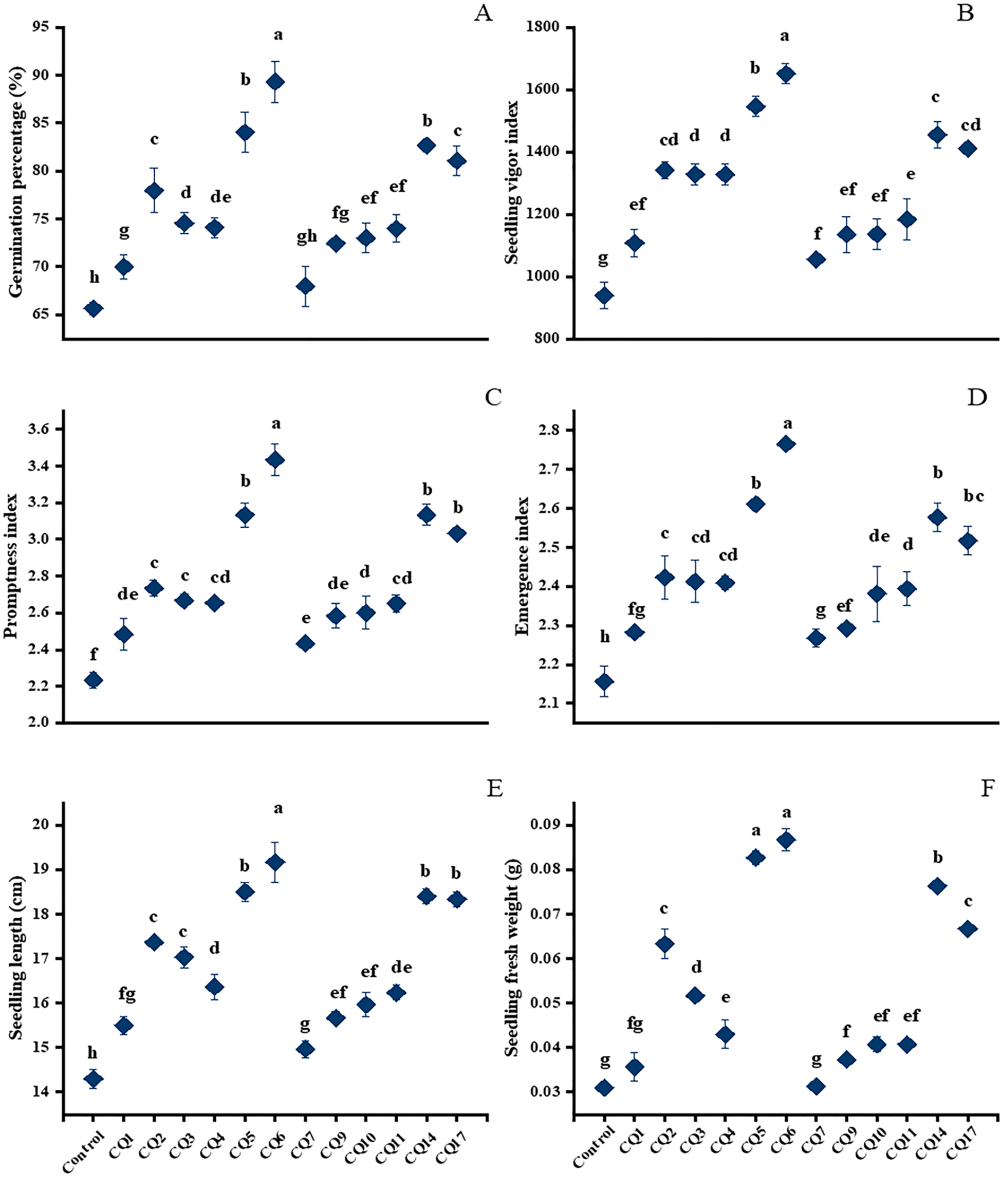
Growth promotion activity of rhizobacteria inoculated tomato seedlings in Petri plate experiment under axenic conditions (**A**) seedling length, (**B**) seedling fresh weight, (**C**) germination percentage, (**D**) seedling vigor index, (**E**) promptness index, (**F**) emergence index. Values are means of three replicates. Values with different letters are significantly different from each other at p ≤ 0.05.

Tomato seeds inoculated with selected PGPR strains showed a higher germination percentage compared to uninoculated control seeds (Supplementary Figs. 1 and 2). The most prominent enhancements were recorded for CQ6 (36%) and CQ5 (26%), respectively, while other strains displayed increases in germination ranging from 4 to 23%, compared to control seeds (Fig. 2A).

Applications of the selected PGPR significantly increased the seedling vigor index (SVI), promptness index (PI) and emergence index (EI). Bacterial isolate CQ6 exhibited the maximum increase, by 72% in SVI, 54% in PI and by 28% in EI, respectively, in comparison with the control. Eight other bacterial strains displayed 23–51% increases in SVI, 19–40% in PI and 11–20% in EI (Fig. 2B–D).

Applications of the selected isolates lead to a significant increase in tomato seedling length and fresh weight compared to the untreated control. CQ6 displayed the maximum enhancement, by 36%, in seedling length and 190% in seedling fresh weight, respectively. The other bacterial strains displayed increases by 14–29% in seedling length and 31–169% in fresh weight (Fig. 2E,F).

### Identification of selected efficient bacterial strains

**Figure 3:**
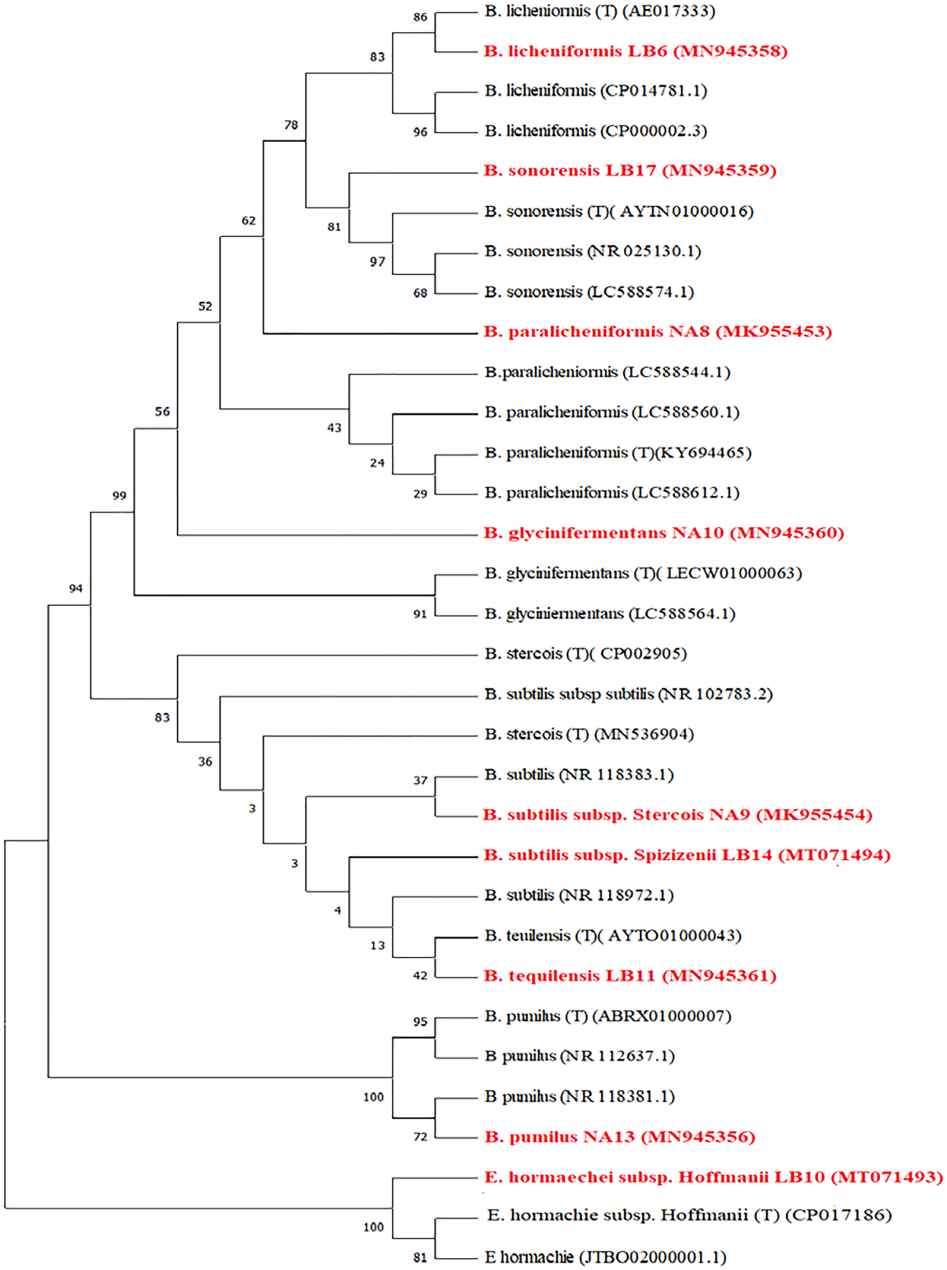
Phylogenetic tree of selected nine bacterial strains inferred using the Neighbor-Joining method. The optimal tree with the sum of branch lengths = 0.35042803 is shown. The percentage of replicate trees in which the associated taxa clustered together in the bootstrap test (1000 replicates) are shown next to the branches. The evolutionary distances were computed using the Maximum Composite Likelihood method and are in the units of the number of base substitutions per site. This analysis involved 32 nucleotide sequences. All ambiguous positions were removed for each sequence pair (pairwise deletion option). There were a total of 1469 positions in the final dataset. Evolutionary analyses were conducted in MEGA X The red colored names are the strains isolated from the rhizosphere of *C. quinoa* in this experiment.

Based on nucleotide identity and phylogenetic analysis of near-complete 16S rRNA gene sequences, the nine most efficient antagonistic isolates were identified, of which eight belonged to the *Bacillus* genus and one from *Enterobacter* (Table 2, Fig. 3). The isolated bacteria were identified as follows (with a similarity index percentage to their respective type strains in parentheses): CQ2, *B. paralicheniformis* (99.6); CQ3, *B. subtilis* subsp*. stercois* (99.9); CQ4, *B. glycinifermentans* (99.6); CQ5, *B. pumilus* (99.7); CQ6, *B. licheniformis* (99.7); CQ10, *E. hormachei* subsp*. hoffmanii* (99.6); CQ11, *B. tequilensis* (100); CQ14, *B. subtilis* subsp*. spizinenii* (100), and CQ17, *B. sonorensis* (99.7).

**Table 2:** Homology of rhizobacterial strains isolated.

NCBI submitted label	Modified label	Homology %	Closest organism	Accession No.
Z1	CQ2	99.63	*B. paralicheniformis*	MK955453
Z2	CQ3	99.92	*B. subtilis* subsp. *stercois*	MK955454
NA10	CQ4	99.65	*B. glycinifermentans*	MN945360
13	CQ5	99.79	*B. pumilus*	MN945356
LB6	CQ6	99.71	*B. licheniformis*	MN945358
LB10	CQ10	99.62	*E. hormachei* subsp. *hoffmanii*	MT071493
LB11	CQ11	100	*B. tequilensis*	MN945361
LB14	CQ14	100	*B. subtilis* subsp. *spizinenii*	MT071494
LB17	CQ17	99.78	*B. sonorensis*	MN945359

### Effects of selected bacterial strains on growth of tomato plants infected with *A. alternata* in pot experiment

From the in vitro experiment, nine bacterial strains were selected based on their antagonistic and plant growth-promoting potential for an *in-planta* experiment. In this pot experiment, *A. alternata* infection significantly reduced all the growth parameters of the tomato plants compared to the uninfected healthy controls. In contrast, plants inoculated with selected bacterial strains along with pathogen infection showed significantly higher root and shoot lengths, as well as root and shoot fresh and dry weights compared to the plants inoculated with *A. alternata* alone (Table 3). *A. alternata* infection reduced tomato root and shoot lengths by 28 and 17%, respectively, compared to the uninfected untreated plants. However, all the plants with bacterial inoculations exhibited significant enhancements in root length ranging from 10 to 263% compared to the uninoculated infected control. Among all the selected PGPRs, *B. licheniformis*, *B. pumilus* and *B. subtilis* subsp*. spizizenii* displayed maximum increases in root length by 263, 245 and 245% and in shoot length by 34, 28 and 30%, respectively, compared to the infected plants without bacterial treatment (Table 3).

**Table 3:** Effect of selected bacterial strains on tomato plant growth following *A. alternata* infection.

Treatments	RL (cm)	SL (cm)	RFW (g)	RDW (g)	SFW (g)	SDW (g)
Control	21.33^e^	49^f^	1.4333^d^	0.6767^c^	9.8333^f^	1.983^g^
*A. alternata*	15.167^f^	43.167^g^	0.4167^e^	0.1767^e^	6.9000^g^	0.8067^h^
*B. paralicheniformis*	44^b^	59.333^c^	1.5667^cd^	0.7133^c^	20.167^cd^	3.5800^d^
*B. subtilis* subsp*. stercois*	35^c^	54.667^d^	1.5500^cd^	0.6467^c^	18.500^d^	3.333^d^
*B. glycinifermentens*	25^d^	50.00^c^	1.4800^d^	0.3207^d^	14.867^e^	2.8167^e^
*B. pumilus*	52.33^a^	60.333^bc^	2.600^b^	1.0400^a^	32.667^a^	5.5637^b^
*B. licheniformis*	55^a^	65.000^a^	2.9000^a^	1.0733^a^	34.667^a^	6.3223^a^
*E. hormachei* subsp*. hoffmanii*	20.333^e^	51.000^ef^	1.4333^d^	0.1957^e^	13.000^e^	2.1007^g^
*B. tequilensis*	21^e^	53.333^de^	1.5100^d^	0.2670^de^	14.000^e^	2.2533^f^
*B. subtilis* subsp*. spizinie*	52.33^a^	62.333^b^	2.5000^b^	1.0400^a^	26.000^b^	5.2277^c^
*B. sonorensis*	44^b^	65.000^a^	1.7000^c^	0.8567^b^	22.000^c^	5.1000^c^

*RL* root length, *SL* shoot length, *RFW* root fresh weight, *RDW* root dry weight, *SFW* shoot fresh weight, *SDW* shoot dry weight.

All the values are the mean of three replicates and bearing different letters in the same column are significantly different at p ≤ 0.05.

*Alternaria alternata* infections significantly reduced root fresh weight (RFW) and root dry weight (RDW) by 243 and 283% and shoot fresh weight (SFW) and shoot dry weight (SDW) by 42 and 146%, respectively, compared to the uninfected control. All the selected PGPRs combined with *A. alternata* infections significantly increased root fresh and dry weights ranging from 51 to 87% and shoot fresh and dry weights ranging from 10 to 85% compared to the untreated infected control. Among all PGPR inoculations, *B. licheniformis, B. pumilus* and *B. subtilis* subsp*. spizizenii* exhibited maximum increases by 85, 84 and 83% in RFW, 84, 83 and 83% increase in RDW, 80, 79 and 73% increase in SFW and 87, 85 and 84% increase in SDW, respectively (Table 3).

### Disease severity index and disease protection

**Figure 4:**
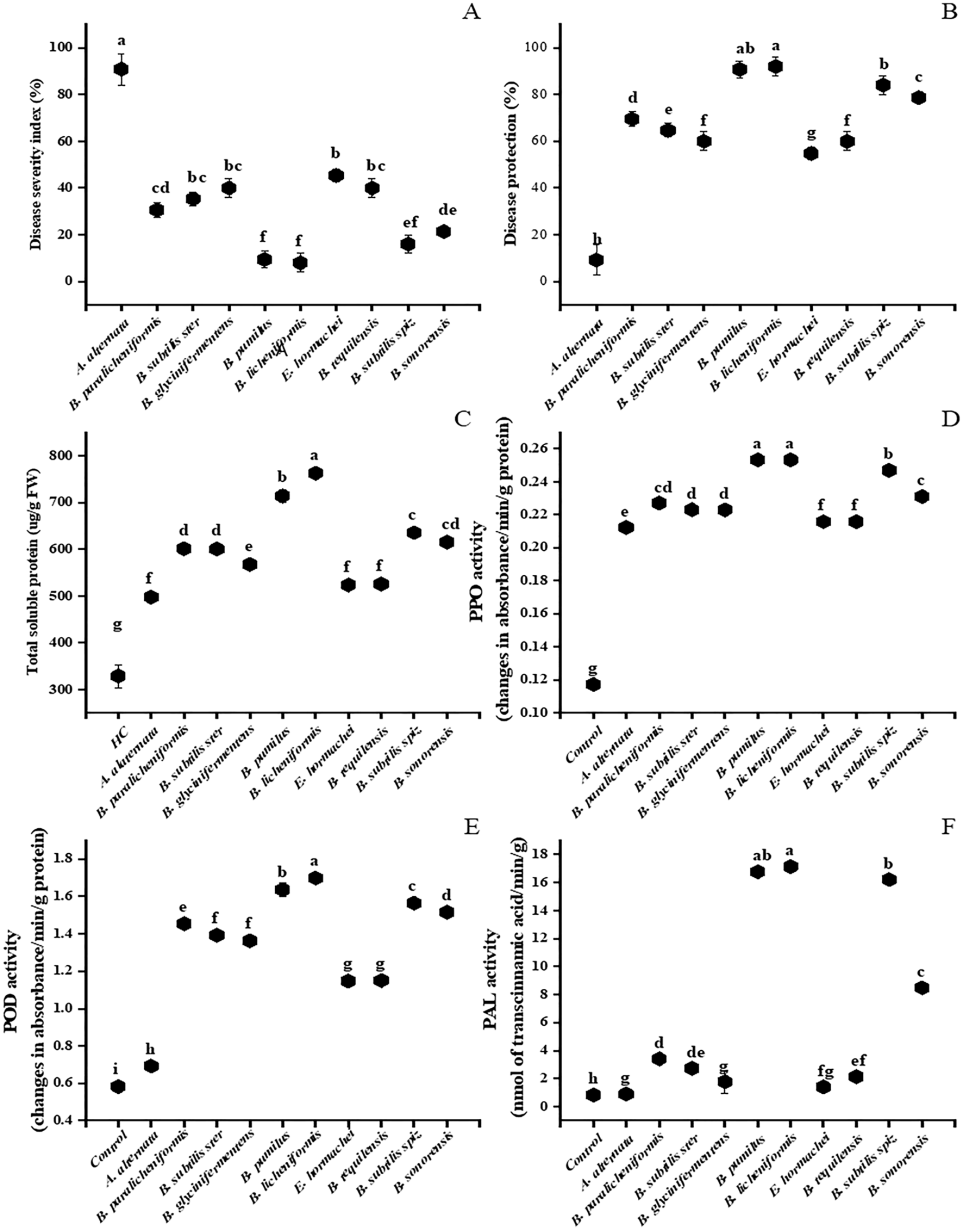
Rhizobacteria-mediated disease protection and induction of antioxidant enzymes in tomato infected with *Alternaria alternata*, (**A**) Disease severity index, (**B**) Disease protection, (**C**) Total soluble protein content (**D**) Polyphenol oxidase (PPO) activity, (**E**) Peroxidase (POD) activity, (**F**) Phenylalanine ammonia lyase (PAL) activity. All values are mean of three replicates. Values with different letters are significantly different from each other at p ≤ 0.05.

All the selected PGPR strains significantly reduced disease severity index and showed significant disease protection against *A. alternata* infection (Fig. 4A,B). Minimum disease severity index and maximum disease reduction/protection was observed in *B. licheniformis* (92%) and *B. pumilus* (90%) followed by *B. subtilis* subsp*. spizizenii* (83%), *B. sonorensis* (76%), *B. paralicheniformis* (66%), *B. subtilis* subsp. *stercois* (61%), *B. glycinifermentans* (56%), *B. tequilensis* (56%) and *E. hormachie* (51%) compared to the untreated infected control.

### Total soluble protein content

Fungal infection enhanced total soluble protein content by 51% at 6 dai compared to the uninfected control. All the PGPR-inoculated plants exhibited increases in protein content compared to the untreated *A. alternata*-infected control. For the PGPR treatments the maximum increases in protein content were observed in *B. licheniformis* and *B. pumilus* by 53 and 43%, respectively, while *B. subtilis* subsp. *spizizenii*, *B. sonorensis*, *B. paralicheniformis*, *B. subtilis* subsp. *stercois*, *B. glycinifermentans*, *B. tequilensis* and *E. hormachie* subsp. *hoffmanii* exhibited 5–28% increases (Fig. 4C).

### Induction of antioxidant defense enzymes

Application of selected PGPR strains induced antioxidant defense enzyme activities in *A. alternata*-infected tomato plants compared to the untreated infected control.

Polyphenol oxidase (PPO) was higher in fungal infected plants by 16% compared to the uninfected control. The selected PGPR strains exhibited greater PPO activity compared to untreated infected control (Fig. 4D). The maximum increase in PPO activity was observed in *B. licheniformis* (17%).

An increase in peroxidase (POD) activity by 45% was observed in *A. alternata*-infected plants as compared to the uninfected control while PGPR applications exhibited even greater enhancement of POD activity compared to untreated fungal infected plants. The maximum increases were observed in plants treated with *B. licheniformis* and *B. pumilus* (59 and 58%, respectively), followed by *B. tequilensis* and *E. hormachie* (40 and 39%, respectively) compared to the untreated infected control (Fig. 4E).

*Alternaria alternata* infections enhanced PAL activity by 8% as compared to healthy plants while PGPR applications following *A. alternata* infections increased PAL activity compared to the fungal infected control. Maximum increase in PAL activity was observed in *B. licheniformis,* as well as *B. pumilus* and *B. subtilis* subsp. *spizizenii* by 95 and 94%, respectively, compared to the untreated infected control (Fig. 4F).

### Differential expression of defense-related genes

**Figure 5:**
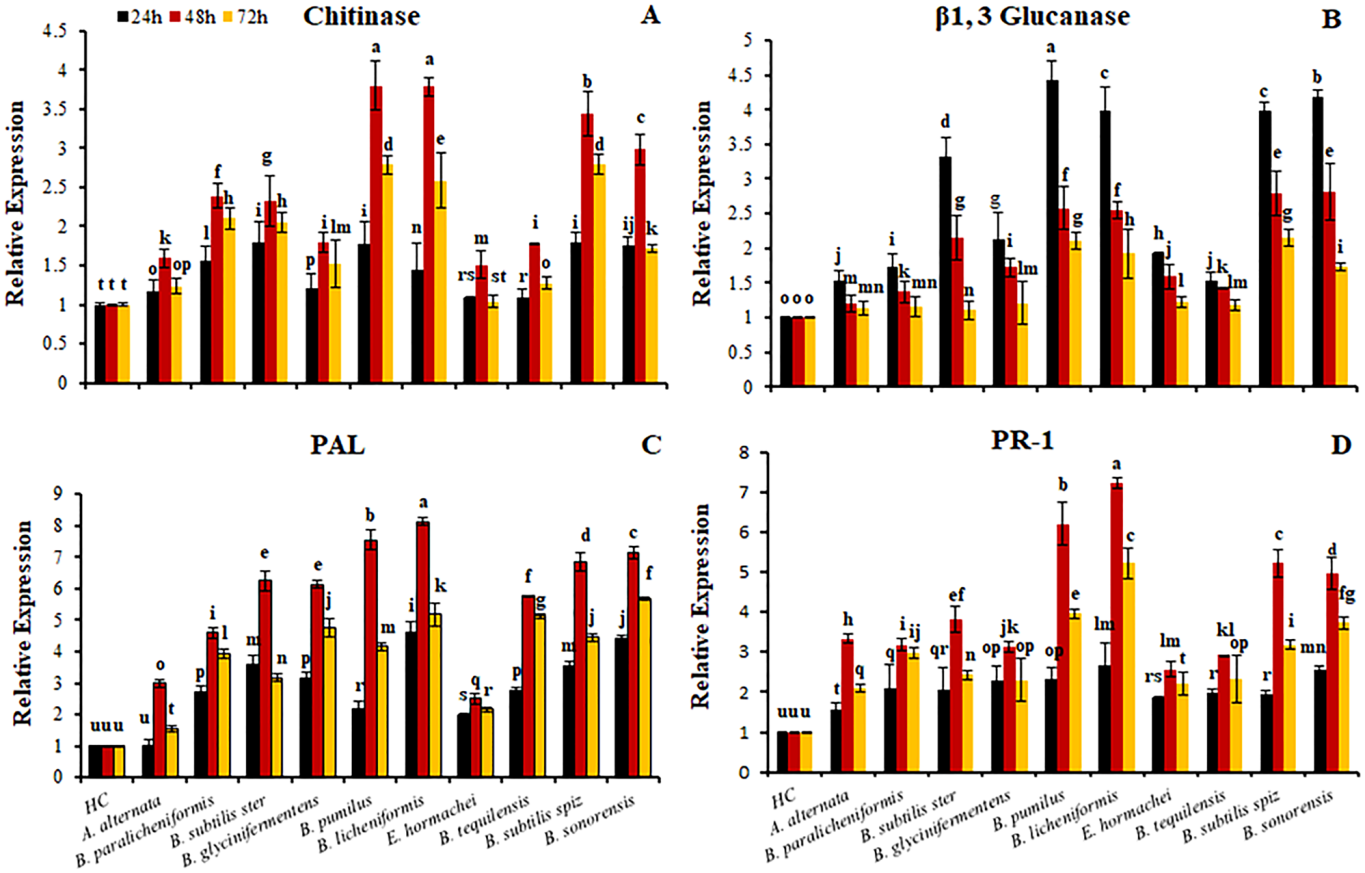
Rhizobacteria-mediated differential expression of defense-related genes in tomato infected with *Alternaria alternata*. All values are means of three replicates.

Upregulation of all the selected defense related genes after fungal inoculation was observed as compared to healthy uninoculated control; however, bacterial application further increased the expression of defense-related genes. The expression level of the acidic chitinase gene was enhanced at 24 h after inoculation and reached a maximum at 48 h with 1.5-fold change with a slight decrease at 72 h. All bacterial treatments further enhanced expression of the acidic chitinase gene with a maximum value of 3.7 after 48 h of infection and maximum fold change was observed by *B. pumilus* and *B. licheniformis* (Fig. 5A).

PR-1 and PAL genes were upregulated by disease induction with a fold change of 1.6 and 1.0 after 24 h of infection. At 48 h maximum expression was observed with a slight decrease at 72 h; however, most of bacterial treatments further enhanced the expression of these genes. Maximum expression was observed by the application of *B. pumilus* and *B. licheniformis* with a fold change of 6.2, 7.3 in PR-1 expression and 7.5, 8.1 in PAL expression (Fig. 5C,D).

*Alternaria alternata* infection also upregulated the expression of the β-1,3 glucanase gene with a fold change of 1.53 at 24 h with a subsequent decrease in expression at 48 and 72 h. *B. pumilus* and *B. paralicheniformis* further enhanced the expression of the β-1,3 glucanase gene with a maximum value of fold change of 4.4 and 3.9 at 24 h post inoculation. A subsequent decrease in expression of the β-1,3 glucanase gene was observed at 48 and 72 h in all the treatments (Fig. 5B).

Gene expression analysis of chitinase, PR-1 and PAL was greatly upregulated at 48 h of *A. alternata* infection in the PGPR inoculated tomato plants while the expression of β-1,3 glucanase was found to be maximum at 24 h of infection. Our results revealed a positive correlation between the expression of these particular genes at 24, 48 and 72 h of infection and significant reduction in *A. alternata* disease incidence (Table 4).

**Table 4:** Pearson correlation coefficients for *A. alternata* disease suppression in tomato plant and expression of defense related genes. Means labeled with ***showed significant positive correlation at p ≤ 0.001, with ** at p ≤ 0.01, and with * at p ≤ 0.05.

Defense gene expression	Correlation co-efficient		
	24 h	48 h	72 h
Chitinase	0.7347**	0.7663***	0.6038*
Β-1,3 Glucanase	0.8351***	0.7005**	0.6662*
PAL	0.6334*	0.7242**	0.3125*
PR-1	0.5593*	0.8448***	0.6040*

## Discussion

The use of rhizosphere bacteria to control pathogens and promote plant growth is advocated in many reports. PGPR help host plants to suppress pathogens as well as absorb mineral nutrients by making them bioavailable^59,60^. PGPR can strengthen plant defense by initiation of induced systemic resistance^61^. The present study focused on the isolation, screening and characterization of rhizospheric bacteria isolated from quinoa (*Chenopodium quinoa*). This ancient grain crop is differentiated from other plants in terms of abiotic stress resistance due to its ability to grow in saline, arid and water-deficient conditions, as well as the regeneration potential of its broken seeds^62^. Microbes belonging to the genus *Bacillus* are known for their vertical transmission from one generation to the other; thus, it can be assumed that the presence of specific microbial diversity associated with quinoa may be a factor in its survival.

In this study, bacterial isolates were screened based on their antifungal potential and nutrient solubilization for plant growth promotion. The screened bacteria were able to solubilize phosphorus and zinc but no potassium solubilization potential was observed in any strain. PGPR are reported to solubilize minerals by liberating organic acids; i.e., gluconic acid, lactic acid, oxalic acid and citric acid^63,64^. These organic acids are responsible for decreasing soil pH, which favors the solubilization of nutrients like zinc and phosphorus, but makes potassium less available^65–67^. The reason for the lack of potassium solubilization observed here may be due to an inate inability of the bacteria to solubilize this nutrient or a pH effect, but further studies are needed to understand this in detail.

Twelve bacteria among the isolated strains effectively inhibited fungal mycelial growth, which may have been due to production of hydrolytic enzymes and siderophores by these strains. Pectinase, cellulase, amylase and siderophore production was observed in these selected strains, which likely enabled the bacteria to inhibit the radial growth of the *A. alternata* mycelia. Previous studies have reported that biocontrol agents are considered good PGPR^68,69^. Proteases, cellulases and other bacterial enzymes secreted exogenously have a role in promoting soil fertility and control of phytopathogen invasion by the mechanism of disintegration of the pathogen membrane (fungal cell wall). Siderophores are key compounds produced by a diverse group of antagonistic PGPR such as *Bacillus*, *Azotobactor*, *Rhizobium* and *Pseudomonas*^70–72^. They impede the proliferation of phytopathogens by sequestering Fe^3+^ in the rhizosphere, making it unavailable to pathogens^15^. Studies have reported that the siderophores produced by biocontrol *Bacillus* stains control diseases caused by *R. solani*, *F. oxysporum*, *S. rolfsii*, *F. solani* and *G. graminis* var. *tritici* and stimulate plant growth^73–76^.

Results obtained from in vitro experiments under axenic conditions revealed the growth-promoting properties of the 12 strains selected above. These bacteria improved the germination percentage, seedling vigor index and emergence index of tomato plants as compared to the control (Fig. 1). Similar findings were reported by different researchers in various crops such as maize^77,78^, wheat^79,80^, canola^81^ and sorghum^82^. The improved growth parameters associated with the bacterial seed treatment may have been due to increased production of phytohormones, such as gibberellins, which promote the synthesis of specific enzymes like α-amylase, which is partly responsible for starch breakdown and plays a role in early growth promotion^77^. Similarly, seedling vigor index improvement may be due to an increase in auxin synthesis^77,83^.

PGPRs have been isolated from the rhizosphere of several plants, including maize^84^, wheat^85^ and rice^86^. However, only a few studies have reported on the PGPR associated with quinoa roots^87,88^. Here, we isolated native PGPR associated with quinoa roots from soils in Pakistan and assessed their role in tomato plant growth and disease resistance. Out of 68 isolates, nine strains (CQ2, CQ3, CQ4, CQ5, CQ6, CQ10, CQ11, CQ14 and CQ17) were characterized for their potential PGPR properties. Genotyping revealed that these strains corresponded to *B. paralicheniformis*, *B. subtilis* subsp*. stercois*, *B. glycinifermentans*, *B. pumilus*, *B. licheniformis*, *E. hormachei* subsp. *hoffmanii*, *B. tequilensis*, *B. subtilis* subsp. *spizinenii* and *B. sonorensis*, respectively.

To resist fungal infections, plants naturally trigger several physiological responses including phytohormone synthesis, nutrient uptake regulation and the production of enzymes for antioxidant defense. Rhizosphere bacteria are known to confer resistance and protection against various plant pathogens^89^; however, very few studies have reported biocontrol agents having antagonistic potential along with plant growth promotion ability. The present study was, therefore, designed to explore the potential of rhizosphere bacteria to control the *A. alternata* pathogen with concurrent stimulatory effects on plant growth. A biocontrol agent having broad-spectrum antagonistic as well as growth promotion ability is more promising under field conditions^90,91^.

Results obtained from the pot experiment here validated the innate biocontrol and biofertilizer potential of selected nine bacterial strains identified. *In planta* evaluation showed that tomato plants infected with *A. alternata* and treated with *B. licheniformis* and *B. pumilus* showed the greatest disease protection and biomass. This response may be attributed to the multifarious potential of both the bacterial agents that combined disease control with plant growth promotion. PGPR application triggered plant immunity through induced systemic resistance (ISR) as this successfully controlled leaf blight disease caused by *A. alternata* in the tomato plants. Disease control activity of these strains may be attributed to the production of antifungal metabolites and the collective action of lytic enzymes and siderophore production. Increased production of these protective molecules by PGPR can result in an enhanced nutrient uptake by host plants infected with a fungal pathogen^69,71^.

*B. licheniformis* and *B. pumilus* inoculations exhibited increases in total soluble protein content and antioxidant defense enzyme activities including POD, PPO and PAL. Increased production of protein supports the increased activity of plant defense mechanisms in generating stress-related enzymes^92^. Increases in PPO and POD activities triggers the biosynthesis of phenolic compounds, which leads to the strengthening of plant defense against pathogen attack, while PAL promotes the activation of phenylpropanoid mechanism to induce the production of phytoalexins, resulting in cell wall lignification and suppression of pathogen attack^93^. Many researchers have reported the ISR potential of PGPRs through increased activities of defense-related enzymes against fungal attack^69,92–94^.

*Alternaria alternata* infection in tomato plants induced the expression of pathogenesis-related proteins including chitinase, β-1,3 glucanase, PR-1 and PAL genes. We found a significant increase in expression of all studied genes after fungal infection at 24 h post inoculation. Transcript abundance continued to increase at 48 h (with the exception β-1,3 glucanase), while after 72 h the expression of all genes started to decline. The PR proteins are small, mostly acidic, resistant to breakdown and most commonly found in intercellular spaces. The activation of these genes is reported to prevent the progress of damage in the plant tissues^95^. The biochemical products of these genes result in activation of defense-related pathways as well as degradation of pathogen cell walls^96^. However, overexpression of the PAL gene is also linked to the strengthening of the plant defense barrier by lignification. Our results showed that tomato plants inoculated with the bacteria *B. pumilus* and *B. paralicheniformis* significantly enhanced the gene expression of chitinase, β-1,3 glucanase, PR-1 and PAL genes at 24, 48 and 72 h post inoculation as compared to untreated infected control. The expression of β-1,3 glucanase has been reported to be maximum at 24 h in wheat infected with *Puccinia triticina*^96^ while maximum expression of PR-proteins, chitinase and PAL has been reported maximum at 48 h post inoculation^97^.

In conclusion, this study adds support to the application of PGPR as a valuable strategy for the improvement of agricultural productivity through enhancement of plant defense against fungal pathogens.

## Supplementary Information

Supplementary Information.

## Data Availability

All data generated or analyzed during this study are included in the supplementary information files.

## References

[1] Glick BR (2012). Plant growth-promoting bacteria: Mechanisms and application. Scientifica..

[2] Ho YN, Mathew DC, Huang CC (2017). Plant-microbe ecology: Interactions of plants and symbiotic microbial communities. Plant Ecol. Traditional Approaches Recent Trends..

[3] Glick BR, Gamalero E (2021). Recent developments in the study of plant microbiomes. Microorganisms.

[4] Smith SE, Smith FA (2011). Roles of arbuscular mycorrhizas in plant nutrition and growth: New paradigms from cellular to ecosystem scales. Annu. Rev. Plant Biol..

[5] Du Y (2019). Comparative genomic analysis of *Bacillus paralicheniformis* MDJK30 with its closely related species reveals an evolutionary relationship between *B. paralicheniformis* and *B. licheniformis*. BMC Genomics.

[6] Oku S, Komatsu A, Tajima T, Nakashimada Y, Kato J (2012). Identification of chemotaxis sensory proteins for amino acids in *Pseudomonas fluorescens* Pf0-1 and their involvement in chemotaxis to tomato root exudate and root colonization. Microbes Environ..

[7] Kumar V, Singh S, Singh J, Upadhyay N (2015). Potential of plant growth promoting traits by bacteria isolated from heavy metal contaminated soils. Bull. Environ. Contam. Toxicol..

[8] Hassani MA, Durán P, Hacquard S (2018). Microbial interactions within the plant holobiont. Microbiome.

[9] Kumar A, Maurya BR, Raghuwanshi R (2014). Isolation and characterization of PGPR and their effect on growth, yield and nutrient content in wheat (*Triticum aestivum* L.). Biocatal. Agric. Biotechnol..

[10] Jasim B, Jimtha CJ, Jyothis M, Radhakrishnan EK (2013). Plant growth promoting potential of endophytic bacteria isolated from *Piper nigrum*. Plant Growth Regul..

[11] Kumar K, Hundal LS (2016). Soil in the city: Sustainably improving urban soils. J. Environ. Qual..

[12] Pastor N, Carlier E, Andrés J, Rosas SB, Rovera M (2012). Characterization of rhizosphere bacteria for control of phytopathogenic fungi of tomato. J. Environ. Manage..

[13] Hammami I, Hsouna AB, Hamdi N, Gdoura R, Triki MA (2013). Isolation and characterization of rhizosphere bacteria for the biocontrol of the damping-off disease of tomatoes in Tunisia. C. R. Biol..

[14] Babu AN, Jogaiah S, Ito SI, Nagaraj AK, Tran LSP (2015). Improvement of growth, fruit weight and early blight disease protection of tomato plants by rhizosphere bacteria is correlated with their beneficial traits and induced biosynthesis of antioxidant peroxidase and polyphenol oxidase. Plant Sci..

[15] Martínez-Hidalgo P, García JM, Pozo MJ (2015). Induced systemic resistance against Botrytis cinerea by Micromonospora strains isolated from root nodules. Front. Microbiol..

[16] Ahmad A, Shafique S, Shafique S, Akram W (2014). *Penicillium oxalicum* directed systemic resistance in tomato against *Alternaria alternata*. Acta Physiol. Plant..

[17] Özer N (2011). Screening for fungal antagonists to control black mold disease and to induce the accumulation of antifungal compounds in onion after seed treatment. Biocontrol.

[18] Riaz HM, Chohan S, Abid M (2021). Occurrence of tomato early blight disease and associated alternaria species in Punjab, Pakistan. J. Anim. Plant. Sci..

[19] Innocenti G, Roberti R, Piattoni F (2015). Biocontrol ability of *Trichoderma harzianum* strain T22 against Fusarium wilt disease on water-stressed lettuce plants. Biocontrol.

[20] Egusa M, Miwa T, Kaminaka H, Takano Y, Kodama M (2013). Nonhost resistance of *Arabidopsis thaliana* against *Alternaria alternata* involves both pre-and postinvasive defenses but is collapsed by AAL-toxin in the absence of LOH2. Phytopathology.

[21] Brandwagt BF, Kneppers TJ, Van der Weerden GM, Nijkamp HJJ, Hille J (2001). Most AAL toxin-sensitive Nicotiana species are resistant to the tomato fungal pathogen *Alternaria alternata* f. sp. *lycopersici*. Mol. Plant Microbe Interact..

[22] Eljounaidi K, Lee SK, Bae H (2016). Bacterial endophytes as potential biocontrol agents of vascular wilt diseases—Review and future prospects. Biol. Control.

[23] Attia MS, El-Sayyad GS, Abd Elkodous M, El-Batal AI (2020). The effective antagonistic potential of plant growth-promoting rhizobacteria against *Alternaria solani*-causing early blight disease in tomato plant. Sci. Hortic..

[24] Guevara-Avendaño E (2018). Antifungal activity of avocado rhizobacteria against *Fusarium euwallaceae* and *Graphium* spp., associated with Euwallacea spp. nr. *fornicatus*, and *Phytophthora cinnamomi*. Antonie Van Leeuwenhoek.

[25] Burkett-Cadena M, Sastoque L, Cadena J, Dunlap CA (2019). *Lysinibacillus capsici* sp. nov, isolated from the rhizosphere of a pepper plant. Antonie Van Leeuwenhoek.

[26] Johnson ET, Dunlap CA (2019). Phylogenomic analysis of the *Brevibacillus brevis* clade: a proposal for three new *Brevibacillus* species, *Brevibacillus fortis* sp. nov., *Brevibacillus porteri* sp. nov. and *Brevibacillus schisleri* sp. nov. Antonie Van Leeuwenhoek.

[27] Dunlap CA (2019). Taxonomy of registered *Bacillus* spp. strains used as plant pathogen antagonists. Biol. Control..

[28] Pane C, Zaccardelli M (2015). Evaluation of Bacillus strains isolated from solanaceous phylloplane for biocontrol of Alternaria early blight of tomato. Biol. Control.

[29] Solanki MK, Yandigeri MS, Kumar S, Singh RK, Srivastava AK (2019). Co-inoculation of different antagonists can enhance the biocontrol activity against *Rhizoctonia solani* in tomato. Antonie Van Leeuwenhoek.

[30] Zhao Z, Liu H, Wang C, Xu JR (2014). Comparative analysis of fungal genomes reveals different plant cell wall degrading capacity in fungi. BMC Genomics.

[31] Chowdappa P, Kumar SM, Lakshmi MJ, Upreti KK (2013). Growth stimulation and induction of systemic resistance in tomato against early and late blight by *Bacillus subtilis* OTPB1 or *Trichoderma harzianum* OTPB3. Biol. Control.

[32] Mahdi I, Fahsi N, Hafidi M, Allaoui A, Biskri L (2020). Plant Growth Enhancement using Rhizospheric Halotolerant Phosphate Solubilizing Bacterium *Bacillus licheniformis* QA1 and *Enterobacter asburiae* QF11 Isolated from *Chenopodium quinoa* Willd. Microorganisms.

[33] Sun Y, Liu F, Bendevis M, Shabala S, Jacobsen SE (2014). Sensitivity of two quinoa (*Chenopodium* quinoa Willd.) varieties to progressive drought stress. J. Agron. Crop Sci..

[34] Schmöckel SM, Lightfoot DJ, Razali R, Tester M, Jarvis DE (2017). Identification of putative transmembrane proteins involved in salinity tolerance in *Chenopodium quinoa* by integrating physiological data, RNAseq, and SNP analyses. Front. Plant Sci..

[35] Karimi, G., Pourakbar, L., Moghaddam, S. S. & Popović-Djordjević, J. Integrated effects of bacteria and fungi biofertilizers on morphological traits, antioxidants indices, and polyphenol compounds of quinoa (*Chenopodium quinoa* Willd.) under salinity condition. (2020). 10.21203/rs.3.rs-123080/v1

[36] Vega-Gálvez A (2010). Nutrition facts and functional potential of quinoa (*Chenopodium quinoa* Willd.), an ancient Andean grain: A review. J. Sci. Food Agric..

[37] Nowak V, Du J, Charrondière UR (2016). Assessment of the nutritional composition of quinoa (*Chenopodium quinoa* Willd.). Food Chem..

[38] Dinesh R (2015). Isolation, characterization, and evaluation of multi-trait plant growth promoting rhizobacteria for their growth promoting and disease suppressing effects on ginger. Microbiol. Res..

[39] Schwyn B, Neilands JB (1987). Universal chemical assay for the detection and determination of siderophores. Anal. Biochem..

[40] Hankin L, Zucker M, Sands DC (1971). Improved solid medium for the detection and enumeration of pectolytic bacteria. Appl. Microbiol..

[41] Schinke C, Germani JC (2012). Screening Brazilian *Macrophomina phaseolina* isolates for alkaline lipases and other extracellular hydrolases. Int. Microbiol..

[42] Nautiyal CS (1999). An efficient microbiological growth medium for screening phosphate solubilizing microorganisms. FEMS Microbiol. Lett..

[43] Bunt JS, Rovira AD (1955). Microbiological studies of some subantarctic soils. J. Soil Sci..

[44] Zhang C, Kong F (2014). Isolation and identification of potassium-solubilizing bacteria from tobacco rhizospheric soil and their effect on tobacco plants. Appl. Soil Ecol..

[45] Naz R (2021). Induction of defense-related enzymes and enhanced disease resistance in maize against Fusarium verticillioides by seed treatment with *Jacaranda mimosifolia* formulations. Sci. Rep..

[46] Nawaz Z, Shu Q (2014). Molecular nature of chemically and physically induced mutants in plants: A review. Plant Genet. Resour..

[47] Russell DW, Sambrook J (2001). Molecular Cloning: A Laboratory Manual.

[48] Tan ZY (1997). Phylogenetic and genetic relationships of *Mesorhizobium tianshanense* and related rhizobia. Int. J. Syst. Evol. Microbiol..

[49] Altschul SF, Gish W, Miller W, Myers EW, Lipman DJ (1990). Basic local alignment search tool. J. Mol. Biol..

[50] Mekadim C (2018). Evaluation of the infB and rpsB gene fragments as genetic markers intended for identification and phylogenetic analysis of particular representatives of the order Lactobacillales. Arch. Microbiol..

[51] Kim M, Chun J (2014). 16S rRNA gene-based identification of bacteria and archaea using the EzTaxon server. Methods Microbiol..

[52] Motallebi P, Niknam V, Ebrahimzadeh H, Enferadi ST, Hashemi M (2015). The effect of methyl jasmonate on enzyme activities in wheat genotypes infected by the crown and root rot pathogen *Fusarium culmorum*. Acta Physiol. Plant.

[53] Jaber LR (2018). Seed inoculation with endophytic fungal entomopathogens promotes plant growth and reduces crown and root rot (CRR) caused by *Fusarium culmorum* in wheat. Planta.

[54] Naz R, Nosheen A, Yasmin H, Bano A, Keyani R (2018). Botanical-chemical formulations enhanced yield and protection against *Bipolaris sorokiniana* in wheat by inducing the expression of pathogenesis-related proteins. PLoS ONE.

[55] Lounaci L, Guemouri-Athmani S, Boureghda H, Achouak W, Heulin T (2016). Suppression of crown and root rot of wheat by the rhizobacterium *Paenibacillus polymyxa*. Phytopathol. Mediterr..

[56] Lowry OH, Rosebrough NJ, Farr AL, Randall RJ (1951). Protein measurement with the Folin phenol reagent. J. Biol. Chem..

[57] Kar M, Mishra D (1976). Catalase, peroxidase, and polyphenoloxidase activities during rice leaf senescence. Plant Physiol..

[58] Peixoto PHP, Cambraia J, Sant’Anna R, Mosquim PR, Moreira MA (1999). Aluminium effects on lipid peroxidation and the actives of enzymes of oxidative metabolism in sorghum. Rev. Bras. Fisiol. Vegetal..

[59] Goswami D, Thakker JN, Dhandhukia PC (2016). Portraying mechanics of plant growth promoting rhizobacteria (PGPR): A review. Cogent Food Agric..

[60] Kim YC, Anderson AJ (2018). Rhizosphere pseudomonads as probiotics improving plant health. Mol. Plant Pathol..

[61] Romera FJ (2019). Induced systemic resistance (ISR) and Fe deficiency responses in dicot plants. Front. Plant Sci..

[62] Lutz, M. & Bascuñán-Godoy, L. The revival of quinoa: a crop for health. Superfood and Functional Food-An Overview and its Utilization to Processed Foods (V. Waisundara, M. Shiomi, Eds.) *In Tech Open*, 37–54, (2017). 10.5772/65451

[63] Han HS, Lee KD (2005). Physiological responses of soybean-inoculation of *Bradyrhizobium japonicum* with PGPR in saline soil conditions. Res. J. Agric. Biol. Sci..

[64] Khan MS, Zaidi A (2007). Synergistic effects of the inoculation with plant growth-promoting rhizobacteria and an arbuscular mycorrhizal fungus on the performance of wheat. Turk. J. Agric. For..

[65] de Werra P, Péchy-Tarr M, Keel C, Maurhofer M (2009). Role of gluconic acid production in the regulation of biocontrol traits of *Pseudomonas fluorescens* CHA0. Appl. Environ. Microbiol..

[66] Lukkani NJ, Reddy ES (2015). Essential nutrients solubilization ability of fluorescent pseudomonads and their multinutrient management. Int. J. Sci. Res..

[67] Parmar P, Sindhu SS (2013). Potassium solubilization by rhizosphere bacteria: Influence of nutritional and environmental conditions. J Microb. Res..

[68] Sharma A, Johri BN (2003). Growth promoting influence of siderophore-producing Pseudomonas strains GRP3A and PRS9 in maize (*Zea mays* L.) under iron limiting conditions. Microbiol. Res..

[69] Lambrese Y (2018). Production of siderophores by the bacterium *Kosakonia radicincitans* and its application to control of phytopathogenic fungi. Bioresour. Technol. Rep..

[70] Ullah H (2020). Multitrait Pseudomonas spp. isolated from monocropped wheat (*Triticum aestivum*) suppress Fusarium root and crown rot. Phytopathology.

[71] Riaz U, Hakeem KR, Dar GH, Mehmood MA, Bhat RA (2021). Plant growth-promoting rhizobacteria (PGPR) as biofertilizers and biopesticides. Microbiota and Biofertilizers.

[72] Zia R (2021). Seed inoculation of desert-plant growth-promoting rhizobacteria induce biochemical alterations and develop resistance against water stress in wheat. Physiol. Plant..

[73] Prasad P, Kumar J (2017). Management of Fusarium wilt of chickpea using brassicas as biofumigants. Legum. Res. Int. J..

[74] Slama HB (2019). Screening for Fusarium antagonistic bacteria from contrasting niches designated the endophyte *Bacillus halotolerans* as plant warden against Fusarium. Front. Microbiol..

[75] Kushwaha P (2020). Bacterial endophyte mediated plant tolerance to salinity: Growth responses and mechanisms of action. World J. Microbiol. Biotechnol..

[76] Gholami A, Shahsavani S, Nezarat S (2009). The effect of plant growth promoting rhizobacteria (PGPR) on germination, seedling growth and yield of maize. World Acad. Sci. Eng. Technol..

[77] Agbodjato NA, Noumavo PA, Adjanohoun A, Agbessi L, Baba-Moussa L (2016). Synergistic effects of plant growth promoting rhizobacteria and chitosan on in vitro seeds germination, greenhouse growth, and nutrient uptake of maize (*Zea mays* L.). Biotechnol. Res. Int..

[78] Mohammad Y (2014). Enhancement of seed germination and seedling vigor of wheat (*Triticum aestivum* L.) following PGPR treatments. Sch. J. Agric. Vet. Sci..

[79] Ilyas N (2020). Exopolysaccharides producing bacteria for the amelioration of drought stress in wheat. Sustainability.

[80] Saeed A (2015). Identification of canola seeds using nearest neighbor and K-nearest neighbor algorithms. Comput. Eng. Intell. Syst..

[81] Widawati, S. Effect of plant growth promoting rhizobacteria and molasses on seed germination and seedling growth of *Sorghum bicolor* L. Moench. in *Proceedings The SATREPS Conference, Indonesia, November. 1*, 94–99. (2017).

[82] Backer R (2018). Plant growth-promoting rhizobacteria: Context, mechanisms of action, and roadmap to commercialization of biostimulants for sustainable agriculture. Front. Plant Sci..

[83] Ranjan A, Mahalakshmi MR, Sridevi M (2013). Isolation and characterization of phosphate-solubilizing bacterial species from different crop fields of Salem, Tamil Nadu, India. Int. J. Nutr. Pharmacol. Neurol. Dis..

[84] Dua S, Sindhu SS (2012). Effectiveness of rhizosphere bacteria for control of root rot disease and improving plant growth of wheat (*Triticum aestivum* L.). J. Microbiol. Res..

[85] Stephen J, Shabanamol S, Rishad KS, Jisha MS (2015). Growth enhancement of rice (Oryza sativa) by phosphate solubilizing Gluconacetobacter sp. (MTCC 8368) and Burkholderia sp. (MTCC 8369) under greenhouse conditions. Biotechnology.

[86] Ortuño N (2013). Enhancing the sustainability of quinoa production and soil resilience by using bioproducts made with native microorganisms. Agronomy.

[87] Yang A (2016). Enhancing salt tolerance in quinoa by halotolerant bacterial inoculation. Funct. Plant Biol..

[88] Compant S, Clément C, Sessitsch A (2010). Plant growth-promoting bacteria in the rhizo-and endosphere of plants: Their role, colonization, mechanisms involved and prospects for utilization. Soil Biol. Biochem..

[89] Ahmad M, Kibret M (2014). Mechanisms and applications of plant growth promoting rhizobacteria: Current perspective. J. King Saud Univ. Sci..

[90] Zhang LN (2019). Consortium of plant growth-promoting rhizobacteria strains suppresses sweet pepper disease by altering the rhizosphere microbiota. Front. Microbiol..

[91] Agrios, G. N. Plant diseases caused by fungi. in *Plant Pathology*, 5th Edn, Vol. 4, 385–614. (Elseiver, 2005).

[92] Senthilraja G, Anand T, Kennedy JS, Raguchander T, Samiyappan R (2013). Plant growth promoting rhizobacteria (PGPR) and entomopathogenic fungus bioformulation enhance the expression of defense enzymes and pathogenesis-related proteins in groundnut plants against leafminer insect and collar rot pathogen. Physiol. Mol. Plant Pathol..

[93] Rais A, Jabeen Z, Shair F, Hafeez FY, Hassan MN (2017). Bacillus spp., a bio-control agent enhances the activity of antioxidant defense enzymes in rice against *Pyricularia oryzae*. PLoS ONE.

[94] Yasmeen T (2020). Biofilm forming rhizobacteria enhance growth and salt tolerance in sunflower plants by stimulating antioxidant enzymes activity. Plant Physiol. Biochem..

[95] Taheri, P. & Tarighi, S. The role of pathogenesis-related proteins in the tomato-Rhizoctonia solani interaction. *J. Bot*. (2012).

[96] Naz R, Bano A, Wilson NL, Guest D, Roberts TH (2014). Pathogenesis-related protein expression in the apoplast of wheat leaves protected against leaf rust following application of plant extracts. Phytopathology.

[97] Nayanakantha, N. M. C., Rawat, S., Ali, S. & Grover, A. Differential expression of defense-related genes in Sinapis alba and Brassica juncea upon the infection of Alternaria brassicae. (2016).

