# Supplementary material for: Rhizosphere bacteria associated with Chenopodium quinoa promote resistance to Alternaria alternata in tomato
Source: Sci Rep. 2022 Nov 8;12:19027. doi: 10.1038/s41598-022-21857-2 (PMC9643462; doi:10.1038/s41598-022-21857-2)
Supplement: Supplementary file 1 — Supplementary Information. [file 41598_2022_21857_MOESM1_ESM.pdf]

***Rhizosphere* bacteria associated with *Chenopodium quinoa* promote resistance to *Alternaria alternata* in tomato**

Sidra Zahoor[1], Rabia Naz[1*], Rumana Keyani[1], Thomas H. Roberts[2], Muhammad Nadeem Hassan[1], Humaira Yasmin[1], Asia Nosheen[1], Saira Farman[3]

[1]Department of Biosciences, COMSATS University Islamabad, Pakistan

[2]School of Life and Environmental Sciences, University of Sydney, Australia

[3]Department of Biochemistry, Abdul Wali Khan University Mardan, Pakistan

Corresponding author*

Rabia Naz

Department of Biosciences, COMSATS University, Park Road, Chak Shahzad, Islamabad, Pakistan

Phone: +92-51-90496086

Email: rabia.naz@comsats.edu.pk

Supplementary Table 1. Colony morphology study of rhizospheric bacteria associated with *C. quinoa*

Bacteria isolates	Size	Elevation	Shape	Texture	Color	Margins	Optical Density	Gram stain
A1	medium	flat	circular	smooth, dry	Off-white	Lobate	opaque	Gram +ve
A2	small	flat	circular	smooth, moist	Off-white	lobate/irregular	opaque	Gram -ve
A3	medium	raised	circular	smooth, moist	Off-white	Entire	opaque	Gram -ve
A4	small	flat	circular	moist, shiny	Off-white	unclear	opaque	Gram +ve
A5	medium	flat	circular	smooth, moist	Off-white	Entire	opaque	Gram -ve
A6	small	flat	irregular	filamentous	Off-white	filamentous	opaque	Gram +ve
A7	large	flat	circular	dry	Off-white	entire to lobate	opaque	Gram -ve
A8	medium	flat	circular	smooth, moist	light yellow	entire	translucent	Gram +ve
A9	punctiform	raised	circular	smooth, moist, slimy	white	entire	translucent	Gram +ve
A10	small	flat	circular	moist, shiny	white	entire	opaque	Gram -ve
A11	medium	flat	irregular	dry, rugose	half white	lobate to filament	opaque	Gram +ve
NA12	small	flat	irregular	moist, shiny	half white	lobate to filament	opaque	Gram +ve
NA13	small	raised	irregular	slimy,mucoid	colourless	irregular	transparent	Gram +ve

NA1	small	raised	circular	moist, shiny	white	entire	opaque	Gram -ve
NA2	small	flat	circular	smooth, moist	half white	entire	transluscent	Gram +ve
NA3	medium	flat	circular	smooth,moist	half white	entire	opaque	Gram +ve
NA4	punctiform	flat	circular	smooth,moist	white	entire	opaque	Gram +ve
NA5	small	flat	circular	moist, shiny	white	entire	transluscent	Gram -ve
NA6	small	convex	circular	bubble shaped	colourless	entire	transparent	Gram -ve
NA7	punctiform	flat	circular	smooth,moist	half white	entire	opaque	Gram +ve
NA8	small	raised	irregular	mucoid,slimy	colourless	flower shaped	transparent	Gram +ve
NA9	small	raised	circular	moist, shiny	light yellow	lobate	opaque	Gram +ve
NA10	small	raised	irregular	bubble shaped	colourless	patterns	transparent	Gram +ve
NA11	medium	flat	circular	smooth, moist	half white	entire	opaque	Gram -ve
LB1	small	flat	spindle	smooth,moist	light yellow	entire	opaque	Gram -ve
LB2	medium	flat	circular	smooth,dry	half white	entire	opaque	Gram +ve
LB3	medium	flat	circular	smooth,moist,shiny	light pink	entire to lobate	opaque	Gram +ve
LB4	small	raised	circular	smooth,moist,shiny	light yellow	entire	opaque	Gram +ve
LB5	small	raised	circular	smooth	white	entire	opaque	Gram +ve
LB6	small	convex	spindle	smooth,mucoid	colourless	mucoid	transparent	Gram +ve

LB7	medium	flat	unclear	smooth	half white	entire	opaque	Gram +ve
LB8	medium	flat	circular	dry	half white	lobate	opaque	Gram +ve
LB9	large	flat	rhizoidal	moist,smooth	half white	rhizoid	opaque	Gram –ve
LB10	small	raised	circular	smooth,moist	half white	entire	opaque	Gram –ve
LB11	small	flat	circular	smooth, dry	half white	unclear	opaque	Gram +ve
LB12	unclear	flat	unclear	dry,rugose	half white	unclear	opaque	Gram +ve
LB13	medium	flat	circular	smooth,moist	white	entire to lobate	opaque	Gram –ve
LB14	small	raised	circular	smooth,moist	half white	entire	opaque	Gram +ve
LB15	small	raised	circular	smooth,moist	half white	entire	opaque	Gram +ve
LB16	medium	convex	irregular	moist,slimy	colourless	bubble	transparent	Gram +ve
LB17	medium	raised	irregular	moist,slimy,bubble	colourless	specific patterns	transparent	Gram +ve
LB18	small	raised	circular	smooth	white	entire	opaque	Gram +ve
FLB1	large	flat	double boundary circular	smooth, moist,slimy	half white	lobate to filament	opaque	Gram +ve
FLB2	small	flat	circular	smooth,moist	white	entire	opaque	Gram +ve
FLB3	small	raised	circular	smooth,moist	white	entire	opaque	Gram –ve

FLB4	medium	raised in middle	irregular	smooth, shiny	yellow	lobate to irregular	opaque	Gram +ve
FLB5	medium	flat	irregular	smooth, shiny	yellow	lobate to irregular	opaque	Gram –ve
FLB6	small	flat	circular	smooth, dry	half white	entire	opaque	Gram +ve
FNA1	small	raised	circular	smooth, dry	yellow	entire	opaque	Gram +ve
FNA2	medium	raised	circular	smooth,moist	yellow	entire	opaque	Gram +ve
FNA3	small	raised	circular	smooth,moist	light orange	irregular	opaque	Gram +ve
FNA4	small	raised	circular	smooth,moist	half white	entire	opaque	Gram –ve
FNA5	medium	flat	irregular	smooth,moist	half white	irregular	opaque	Gram +ve
FNA6	small	flat	circular	smooth,moist	half white	lobate	opaque	Gram +ve
FNA7	small	raised	circular	smooth,moist	half white	entire	opaque	Gram +ve
FNA8	medium	flat	filamentous	dry, smooth	half white	filamentous	opaque	Gram +ve
FNA9	small	raised	circular	moist	half white	entire	opaque	Gram +ve
FNA10	medium	flat	circular	moist	half white	entire	opaque	Gram +ve
FNA11	small	flat	circular	moist	light yellow	entire	opaque	Gram –ve

Supplementary Table 2. Antagonistic potential of isolated rhizospheric bacteria associated with *C. quinoa* from the selected locations against *A. alternata*

**Antagonistic potential of bacteria isolated from Khanewal region against *A. alternata***			**Antagonistic potential of bacteria isolated from Faisalabad region against *A. alternata***		
Bacterial strains	Mycelial growth (cm)	Percent inhibition (%)	Bacterial strains	Mycelial growth (mm)	Percent inhibition (%)
A1	3.5	62	LB10	1.75	80
A2	6.5	31	LB11	1.15	88
A3	2.5	74	LB12	3.7	60
A4	4.2	56	LB13	6.5	25
A5	5.2	42	LB14	0.2	98
A6	3.2	64	LB15	2.6	70
A7	3.15	63	LB16	2.7	69
A8	3.7	60	LB17	0.2	98
A9	3.9	58	LB18	5.2	30
A10	3.15	63	FLB1	5.75	36
A11	6.5	25	FLB2	3.75	58
NA12	4.5	49	FLB3	3.2	64
NA13	0	100	FLB4	2.4	73
NA1	4	55	FLB5	2.55	75
NA2	3.7	60	FLB6	7.2	21
NA3	3.15	63	FNA1	3.75	59

NA4	3.15	63	FNA2	3.7	60
NA5	3.7	60	FNA3	5.3	40
NA6	6.85	31	FNA4	5.6	37
NA7	2.1	77	FNA5	2.3	75
NA8	1.2	87	FNA6	2.3	75
NA9	1.2	87	FNA7	2.4	74
NA10	1.2	87	FNA8	4.3	50
NA11	7.7	14	FNA9	3.9	58
LB1	2.8	68	FNA10	3.7	60
LB2	5.4	40	FNA11	4.7	47
LB3	2.6	70			
LB4	7	29			
LB5	6.8	32			
LB6	0	100			
LB7	0	100			
LB8	2.65	69			
LB9	0	100			

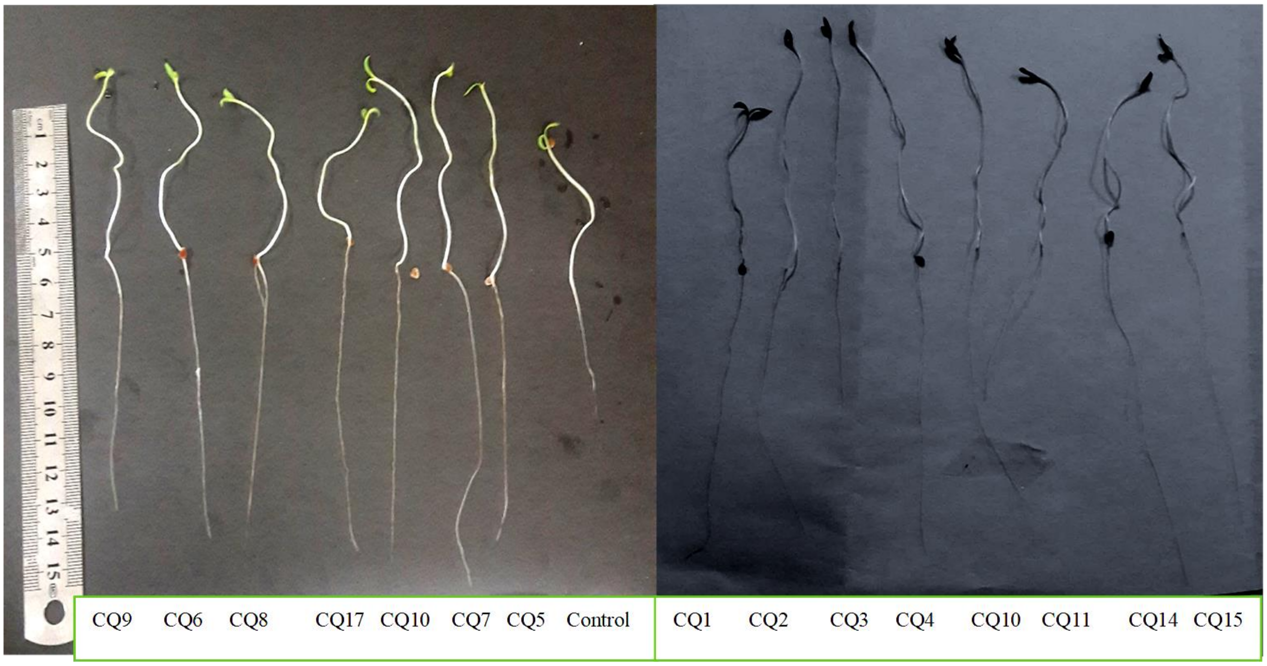

Supplementary Figure 1. Effect of selected bacterial strains on tomato plant growth in Petri plate experiment under axenic conditions

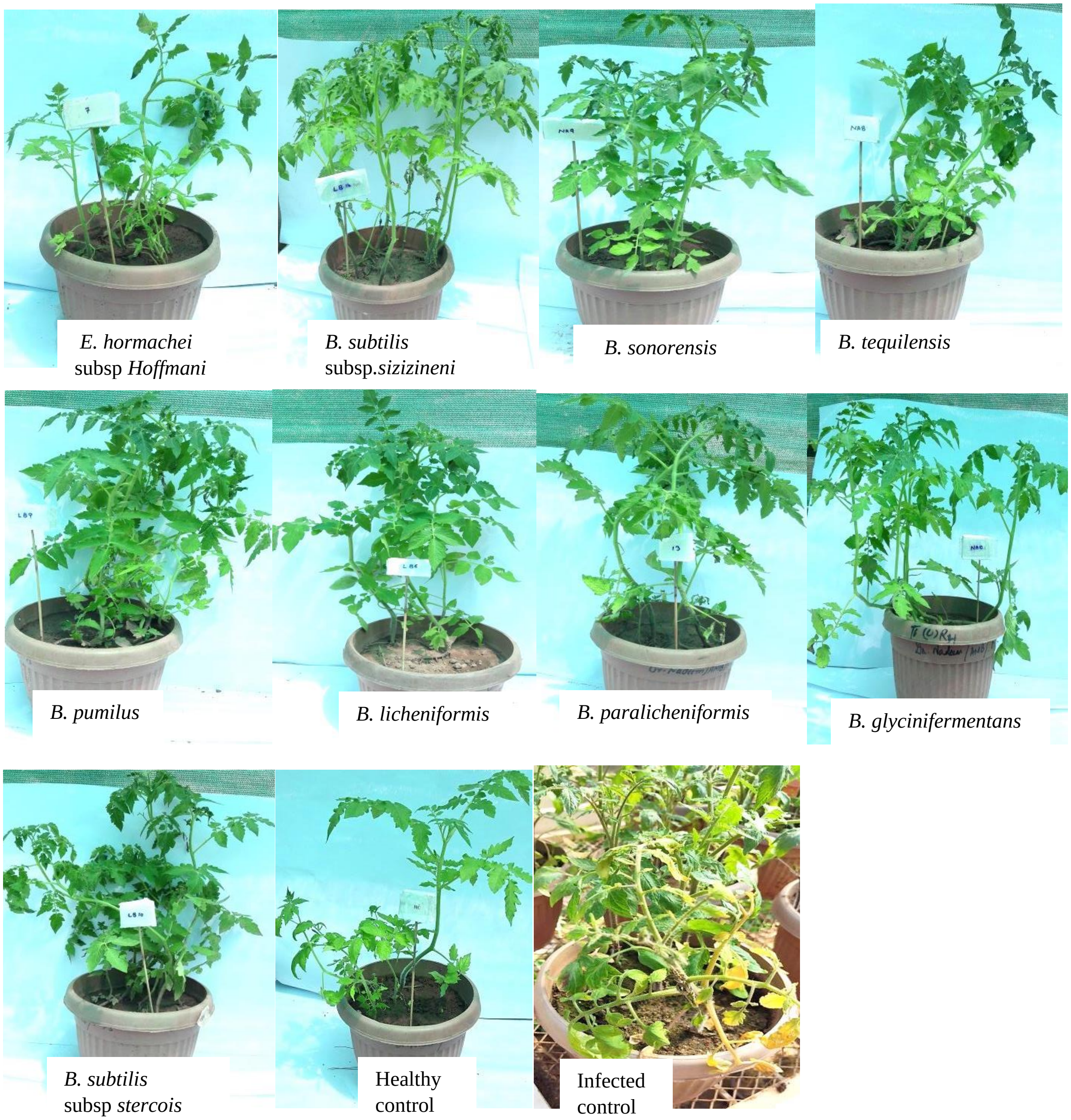

Supplementary Figure 2. Effect of selected bacterial strains on tomato plant following *A. alternata* infection in pot experiment

Supplementary Table 3: Defense related genes used in RT-qPCR with their primers

**Gene**	**Forward primer (5'-3)**	**Reverse primer (5'-3')**	**Tm (ºC)**	**Reference**
Chitinase	CAAGCAGCTAATCGCCTC	CGATCCTGAACCCTGTTAT	56.1;55.2	Jun Cao and Xiaona Tan 2019
β 1,3 Glucanase	GCGGTGTTCAGCCTGGATG	AGCATGAGCAAGAAGTATGTTGTG	61.7;61.8	Murugesan Chandrasekaran & Se Chul Chun 2016
PAL	CGCTATGCTCTCCGAACATCTC	ATTCACCGAGTTAATCTCCCTCTC	61.7;63.5	Murugesan Chandrasekaran & Se Chul Chun 2016
PR-1	TAGTCTGGCGCAACTCAGTC	TGCAAGAAATGAACCACCAT	60.5;54.3	Tornero et al 2007
